# Risk factors for hospitalisation in community-dwelling pre-frail and frail older people: results of a longitudinal study

**DOI:** 10.1186/s12877-024-05458-4

**Published:** 2024-10-19

**Authors:** M. G. A. M. van der Velde, L. P. M. Op Het Veld, E. van Rossum, M. A. C. Jansen, H. R. Haak, M. N. T. Kremers

**Affiliations:** 1 Department of Internal Medicine, Máxima Medical Center Veldhoven, De Run 4600, Veldhoven, 5504 DB The Netherlands; 2 Department of Health Services Research, CAPHRI School for Public Health and Primary Care, Aging and Long Term care Maastricht, Maastricht, The Netherlands; 3 Department of Healthcare Biometrics, Zuyd University of Applied Sciences, Heerlen, The Netherlands; 4 Research Centre on Community Care, Zuyd University of Applied Sciences, Heerlen, The Netherlands; 5 Network Emergency Care Brabant, Tilburg, The Netherlands; 6 Emergency Department, Erasmus MC, Rotterdam, The Netherlands

**Keywords:** Older people, Hospitalisation, Frailty, Community-dwelling

## Abstract

**Background:**

Older adults account for a large proportion of hospital admissions. In this study we aim to bridge a gap between medical and psychosocial factors in predicting hospitalisation.

**Methods:**

Demographic and social characteristics of community-dwelling pre-frail and frail older people were collected by questionnaires every six months during a two year follow-up. Hospital admission within this period was dichotomised as yes/no. To define pre-frailty and frailty the Fried frailty criteria were used. Analysis of risk factors for hospitalisation was performed using multivariable logistic regression.

**Results:**

Hospitalised participants (*n* = 1803) were more often male and frail in comparison to not-hospitalised participants. They also experienced more chronic diseases (54.5% ≥ 4 chronic diseases), poorer self-perceived health (SPH) (76.4% fair to very poor) and lack of informal care (20.1%). In multivariable logistic regression male gender (Odds ratio (OR) 1.65, *p* < 0.001), frailty (vs. pre-frailty) (OR 1.66, *p* = 0.002), reporting lower SPH (OR 3.12, *p* < 0.001) and lacking informal care (OR 1.69, *p* < 0.001) showed significant associations with hospital admission. Subgroup analysis of pre-frail and frail participants, showed consistent associations between male gender (respectively OR 1.61, *p* < 0.001 ; OR 1.72, *p* = 0.085), lower SPH (OR 2.23, *p* = 0.001; OR 31.16, *p* < 0.001), lack of informal care (OR 1.64, *p* = 0.005; OR 2.63, *p* = 0.012) and hospitalisation.

**Conclusion:**

Frailty, male gender, lower SPH and lack of informal care are risk factors for hospitalisation within community-dwelling older people, showing the need of a holistic approach to possibly prevent hospitalisation. Further research should focus on evaluating individual factors for hospitalisation, particularly targeting pre-frail individuals.

**Supplementary Information:**

The online version contains supplementary material available at 10.1186/s12877-024-05458-4.

## Key points

- Frail older adults are at higher risk for hospitalisation compared to pre-frail.

- Associations with hospitalisation: male gender, frailty, lower SPH, no informal care.

- Psychosocial factors emerge as important determinants for hospitalisation.

- Understanding medical and psychosocial factors of frailty is key for effective care planning.

**Supplementary Information:**

The online version contains supplementary material available at 10.1186/s12877-024-05458-4.

## Introduction

Older adults, often with ageing-related chronic conditions, utilise healthcare services more often than younger populations and account for a large proportion of hospital admissions [1, 2]. Hospitalisation is a critical event in the life of older adults. Hospitalised adults face not only stress of the precipitating illness or injury, but also a challenging hospital environment [3, 4]. Whilst hospitalised, some recover well from acute illness, others experience a downward spiral consisting of delirium, falls, functional decline, institutionalisation or death [4–6]. The varying outcomes can be related to the great variation of frailty in older adults.

Frailty is a geriatric syndrome characterised by age-associated declines in physiologic reserve and function across multi-organ systems [7, 8]. It is often defined using the Fried Frailty Criteria, which consists of weight loss, exhaustion, low physical activity, slowness and weakness. The sum of these criteria categorises individuals into one of three frailty stages: not frail (score 0), pre-frail (score 1–2) or frail (score 3–5) [7]. Frailty leads to increased vulnerability for adverse health outcomes and increased utilisation of health care services [9]. However, not every individual classified as frail encounters negative health outcomes, and conversely there are individuals deemed not-frail who do encounter adverse health outcomes despite lacking apparent risk factors. To identify older pre-frail and frail adults who are at risk for adverse health outcomes, it is important to know which individual factors predict these outcomes.

Literature regarding health care use and hospitalisation is mainly focused on medical and health-based factors, such as chronic diseases, medication use and functional limitations [10–12]. Nevertheless, in a frail older population these factors may not be that discriminative due to evident correlation of these factors with the presence of frailty. Furthermore, the concept of frailty itself has gained increasing recognition as a significant predictor for hospitalisation in community-dwelling older adults [13].

This study aims to bridge the gap between medical and psychosocial factors in predicting hospitalisation by identifying these factors within pre-frail and frail individuals. By highlighting disparities between hospitalised pre-frail and frail individuals, we seek to gain insights into both risk and protective factors beyond frailty itself. These insights could inform the development of targeted interventions to prevent hospital admissions by addressing both medical and psychosocial factors.

## Methods

### Study design

This study is based on data collected from an observational cohort which was conducted in Limburg, a province in the Netherlands. Every four years the Community Health Services sends out an extensive questionnaire, the Health monitor, to community dwelling older people aged 55 years and over. This monitor facilitates health care policies informed by the characteristics and needs of this large sample. It comprises standard inquiries about demographic characteristics, chronic diseases, use of health care services and informal care along with items about social, psychological and physical functioning. In 2012 the Fried frailty criteria (weight loss, exhaustion, low physical activity, slowness and weakness) were also included. The frailty criteria were asked as a self-report questionnaire. Previous publications have provided full details on formulation of the used self-reported questions for assessing frailty in this cohort [14]. The sum of the Frailty criteria classifies people into one of three frailty stages: not frail (score 0), pre-frail (score 1–2) or frail (score 3–5).

### Procedure and participants

**Fig. 1:**
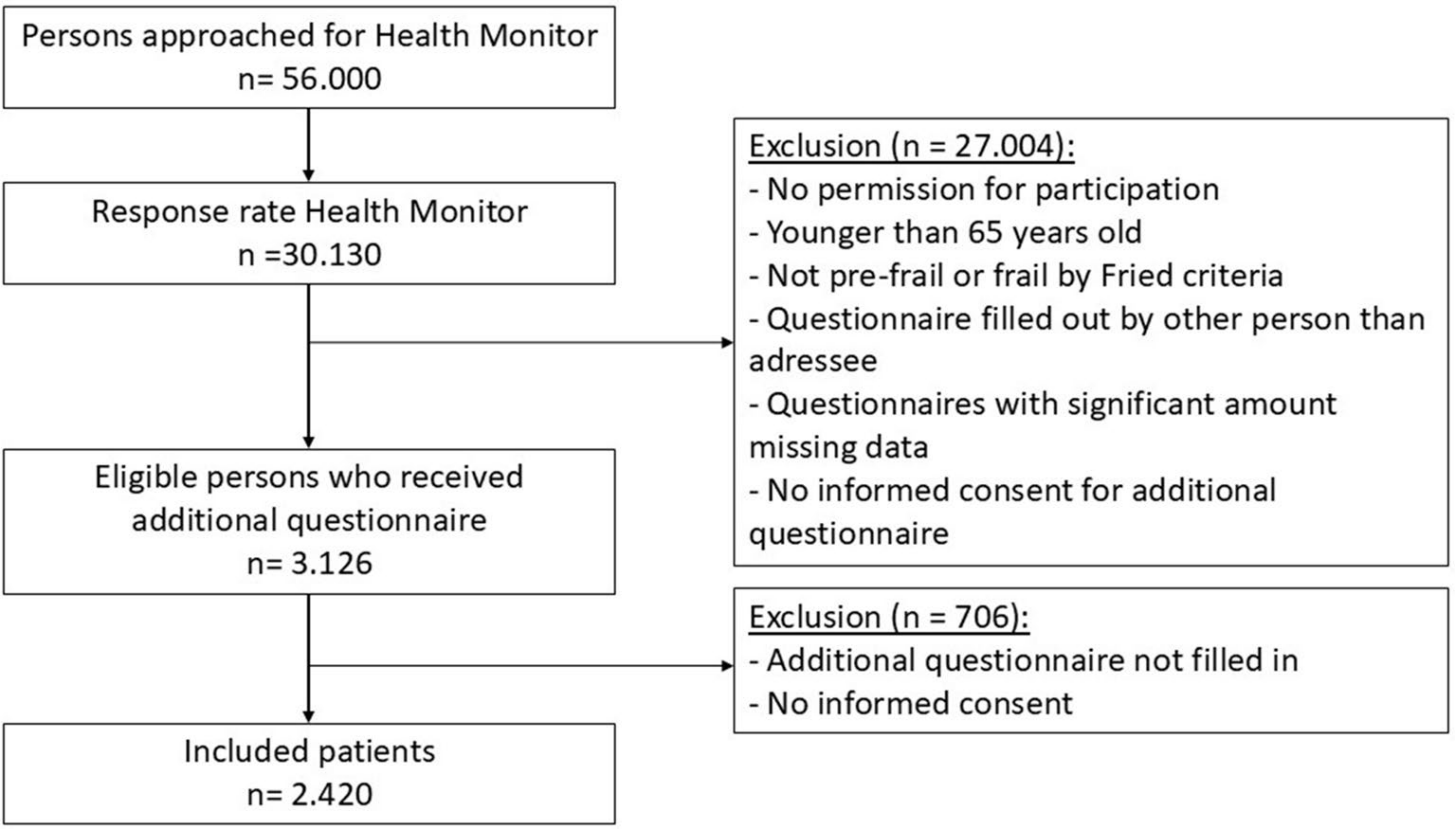
Flowchart for participant-inclusion

The Health Monitor was sent to 56.000 community-dwelling persons (55 + years) in 2012. Response was received from 30.130 persons (53.8%) (Fig. 1). Respondents who were at least 65 years old, gave their informed consent for participation and were considered pre-frail or frail individuals (according to Fried’s frailty criteria) were asked to participate in the study, resulting in 3.126 persons. The selection of this cohort is described in detail in a previous study [14]. These persons were send an additional questionnaire to obtain further baseline data and informed consent for follow-up. The cohort presented in this study comprised the remaining 2.420 persons who completed the questionnaires and provided informed consent. Throughout the two-year study period, participants were asked to complete a questionnaire every six months, which included questions about healthcare use and adverse outcomes. Individuals who died, relocated to a nursing home or explicitly stated their discontinuation of participation were considered as drop-out throughout the duration of the study.

### Data collection

We collected the following demographics and characteristics at baseline from the questionnaires: age, gender, body mass index (BMI, calculated as weight in kilograms divided by height in meters squared), chronic diseases, marital status, living arrangements, use of alcohol, smoking and frailty status according to Fried. In addition we retrieved baseline data regarding self-perceived health, socio-economic status and social network.

Chronic diseases were measured by asking participants whether or not they suffered from one or more of the following chronic diseases: diabetes, stroke/cerebral haemorrhage/cerebral infarction, myocardial infarction, other cardiac diseases, cancer, asthma, chronic obstructive pulmonary disease (COPD), peripheral vascular disease, hypertension, hip or knee arthrosis, chronic joint inflammation or back problems (including hernia).

Self-perceived health (SPH) is a simple and pragmatic tool that is used to measure overall health status. In a single question, “how is your health in general?”, with preset responses on a 5 point Likert scale (in this study; very poor, poor, fair, good, very good), SPH signifies an individual’s subjective assessment of their own health status [15]. SPH was categorised into three categories: very poor-poor, fair and good-very good.

Education was categorised in low, middle and high. The lower category comprises no education, completion of primary school or pre-vocational secondary education. The middle category includes upper secondary education, vocational training and specialist education. The high category included individuals with higher education degrees such as bachelor’s, master’s, and doctoral degrees.

Disposable income per person, adjusted for variations in family composition, was obtained from Statistics Netherlands. Individuals were classified into one of five income groups ranging from low to high income.

Social network is defined consisting of three variables. The type of social network is measured at baseline by using an 8-item questionnaire by Wenger et al. [16]. The results classify people into five types of support networks: family dependent, private restricted, locally integrated, local self-contained and wider community focused. The family dependent and private restricted networks are characterised by a limited number of people available to provide support, whereas locally integrated and wider community focused networks are larger. These limited networks correspond with the absence of availability of informal care [17].

Availability of informal care was assessed by one question at baseline ‘Suppose you got the flu and you had to stay in bed for a couple of days. Is there someone who could take care of you?’, results were dichotomised into ‘yes’ and ‘no’.

Lastly, loneliness was measured at baseline by using De Jong-Gierveld Loneliness Scale [18]. This is a 11-item scale, which allows participants to choose from three answer choices: yes, more or less or no. A higher score indicates more feelings of loneliness.

All collected data were obtained through self-report measures. Participant privacy was ensured by pseudonymisation of the data by replacing all identifying variables with a unique study participant code.

### Outcome measurements

The primary outcome was hospitalisation at any time in the follow-up period of two years, as measured every six-months by the questionnaire. Valid cases among respondents were defined as those with either no missing data or at least one self-reported hospitalisation. Participants with no confirmed hospitalisation throughout the answered questionnaires and missing data at one of the follow-up intervals, were classified as not-valid cases, as the occurrence of hospitalisation could neither be confirmed nor ruled out. Within the valid cases two groups were established: persons who reported at least one admission and those who did not. Outpatient clinic visits or emergency department visits without subsequent hospital admission were not included.

### Statistical analysis

Baseline characteristics of the respondents were analysed using descriptive statistics. Continuous data were presented as mean and standard deviation (SD) or median and interquartile range (IQR), nominal data were presented as total amount and percentage. First, we examined the differences in baseline characteristics between pre-frail and frail participants to better interpret the results of the key analysis (hospitalised versus not-hospitalised participants). P-values were calculated using Pearson’s chi-square or Fisher’s exact test for categorical variables, and the independent-samples t-test or Mann-Whitney U test for continuous variables. In the key analysis, we compared the baseline characteristics between the hospitalised and not-hospitalised groups. We conducted a subgroup analysis to examine disparities between pre-frail individuals who were hospitalised and those who were not, as well as between frail individuals who were hospitalised and those who were not.

The risk factor analysis adhered to the general guideline of including one predictor variable for every 10 events when studying the data. If the number of predictors surpassed the quotient of hospitalisations divided by 10, predictors were selected based on their associated risk estimates in univariable analysis. Univariable logistic regression assessments were conducted independently for each potential prognostic variable and the outcome, without adjustments for potential confounders. The principal analysis entailed multivariable backward stepwise logistic regression (0.05 enter and 0.10 exit), with hospitalisation as the outcome variable. All variables were initially included in the model, and subsequently, through the iterative exclusion of independent variables with the least contribution to the model (identified by the largest p-value), the optimal subset of predictors was determined. No adjustments were made for potential confounders. A significance level of 5% was used for all statistical tests. Statistical analyses were performed using the software Statistical Package for the Social Sciences (SPSS), version 27 (IBM Corp, New York, USA).

### Ethical principles

The study was based on the principles of the Declaration of Helsinki. All participants provided informed consent. The study was approved by the medical ethical committee of Zuyderland and Zuyd University of Applied Sciences (METC Z, 12-N-129).

## Results

### Data collection

Out of the 3,126 eligible individuals for the study, 706 participants (22.6%) did not respond to the additional questionnaires. Since data on these participants are not available, it is not possible to determine whether their absence is random. Therefore, we must classify these participants as missing not at random.

**Fig. 2:**
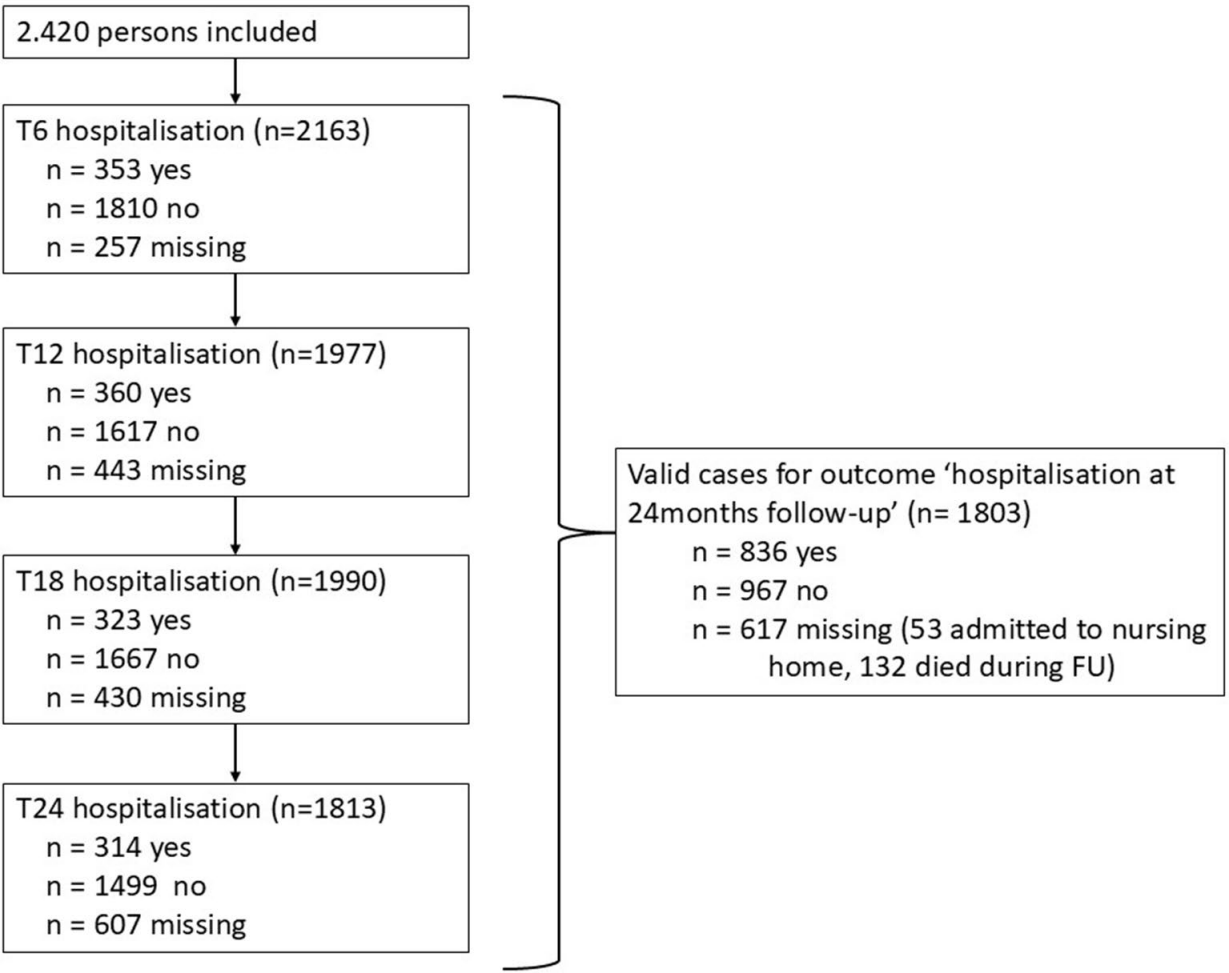
Flow-chart for valid cases for outcome ‘hospitalisation at 24 months’

All analyses were conducted based on valid cases (Fig. 2). Of the 2.420 participants included in the study, 1.803 participants (74.5%) were valid cases for the outcome hospitalisation at 24 months follow-up. Of the total number of missing cases (*n* = 617, 25.5% of the population), about one third can be explained by participants who moved to a nursing home (*n* = 53) or died during follow-up (*n* = 132). The group with valid data was compared to the group with missing data on baseline characteristics using chi-square and Mann-Whitney tests. Participants in the group with missing data were significantly older, more often frail, less educated and more often living alone.

### Baseline characteristics

#### Comparison of frail versus pre-frail participants

The baseline characteristics of the participants are shown in Table 1. Frail participants were compared to pre-frail participants. Similarities were observed in BMI, marital status, household size, and living situation. However, frail participants were older (77.6 years vs. 75.4 years, *p* < 0.001) and had a higher smoking rate (16.3% vs. 11.3%, *p* = 0.011), but lower alcohol consumption (40.1% vs. 74.1%, *p* < 0.001). Frail individuals showed higher rates of various chronic conditions, including diabetes (28.9% vs. 21.0%, *p* = 0.002), stroke (21.1% vs. 13.7%, *p* < 0.001), myocardial infarction (27.8% vs. 14.8%, *p* < 0.001), peripheral vascular disease (25.8% vs. 15.0%, *p* < 0.001), asthma/COPD (28.2% vs. 20.6%), *p* = 0.003) and joint diseases (e.g. chronic joint inflammation 40.6% vs. 24.9%, *p* < 0.001), compared to pre-frail individuals (Table S1). Education levels differed, with frail participants achieving lower education (5.5% vs. 15.4%, *p* < 0.001). Income levels also varied, with more frail participants earning less than pre-frail participants (46.4% earning max €19.400 vs. 33.3%, *p* < 0.001). SPH was poorer among frail participants (5.4% rated as very good or good vs. 39.0%, *p* < 0.001), and they reported higher loneliness scores (median of 5 vs. 3, *p* < 0.001) and hospitalisation rates (61.7% vs. 42.7%, *p* < 0.001).

**Table 1:** Baseline characteristics: frail versus pre-frail participants

		Total *n* = 1803	Frail *N* = 347	Pre—frail *N* = 1456	*p*-value
Age, years		75.8 ± 6.5	77.6 ± 6.8	75.4 ± 6.4	< 0.001
Gender	Male	707 (39.2%)	125 (36.0%)	582 (40.0%)	0.176
	Female	1096 (60.8%)	222 (64.0%)	874 (60.0%)	
BMI (kg/m^2^), continuous		26.8 (24.1–29.5)	27.1 (24.2–30.0)	26.8 (24.0–29.4)	0.093
Marital status	Married/living together	526 (30.1%)	112 (33.4%)	414 (29.3%)	0.142
	Unmarried/divorced/widowed	1220 (69.9%)	223 (66.6%)	997 (70.7%)	
Smoking	Yes	218 (12.1%)	56 (16.3%)	162 (11.3%)	0.011
	No	1558 (86.4%)	287 (83.7%)	1271 (88.7%)	
Alcohol	Yes	1234 (71.5%)	194 (59.9%)	1040 (74.1%)	< 0.001
	No	493 (28.5%)	130 (40.1%)	363 (25.9%)	
Number of chronic diseases	0 or 1	288 (16.0%)	25 (7.2%)	263 (18.1%)	< 0.001
	2 or more	1515 (84.0%)	322 (92.8%)	1193 (81.9%)	
Level of education	High	232 (13.5%)	18 (5.5%)	214 (15.4%)	< 0.001
	Medium	1148 (66.7%)	202 (61.6%)	946 (67.9%)	
	Low	342 (19.9%)	108 (32.9%)	234 (16.8%)	
Income	> €31.000	200 (11.1%)	19 (5.5%)	181 (12.4%)	< 0.001
	Max €31.000	310 (17.2%)	48 (13.8%)	262 (18.0%)	
	Max €24.000	458 (25.4%)	78 (22.5%)	380 (26.1%)	
	Max €19.400	646 (35.8%)	161 (46.4%)	485 (33.3%)	
	Max €15.200	188 (10.4%)	41 (11.8%)	147 (10.1%)	
Household size		2 (1–2)	2 (1–2)	2 (1–2)	0.078
Living situation	Not living alone	645 (36.8%)	197 (59.2%)	910 (64.1%)	0.091
	Living alone	1107 (63.2%)	136 (40.8%)	509 (35.9%)	
Self-perceived health	Very good or good	572 (32.6%)	18 (5.4%)	554 (39.0%)	< 0.001
	Fair	962 (54.8%)	196 (58.7%)	766 (53.9%)	
	Poor or very poor	221 (12.6%)	120 (35.9%)	101 (7.1%)	
Social network type (%)	Private restricted	157 (9.5%)	48 (15.2%)	109 (8.2%)	< 0.001
	Family dependent	359 (21.8%)	77 (24.4%)	282 (21.2%)	
	Locally integrated	606 (36.8%)	100 (31.6%)	506 (38.0%)	
	Local self-contained	401 (24.4%)	73 (23.1%)	328 (24.7%)	
	Wider community focused	123 (7.5%)	18 (5.7%)	105 (7.9%)	
Availability of informal care, no		297 (16.5%)	80 (23.1%)	217 (14.9%)	< 0.001
Loneliness-scale, de Jong-Gierveld		3.9 ± 3.6	5 (2–8)	3 (0–6)	< 0.001
Hospitalision in follow-up of 24 months, yes		836 (46.4%)	214 (61.7%)	622 (42.7%)	< 0.001

BMI: Body Mass Index. Data are reported as number of participants (%), mean ± standard deviation or median (Q1-Q3). Percentages were computed relative to the total number of participants in the presented column. Some baseline data was missing: BMI 5.2%, marital status 3.2%, household size 2.8%, education 4.5%, alcohol 4.2%, smoking 1.5%, SPH 2.7%, loneliness 4.6%

#### Comparison of hospitalised versus not-hospitalised participants

The baseline characteristics of the participants are shown in Table 2. Hospitalised participants were compared to not-hospitalised participants. Similarities in age, BMI, marital status, alcohol intake and social network types were observed. Hospitalised participants were more frequently male (45.7% vs. 33.5%, *p* < 0.001), frail (74.4% vs. 13.8%, *p* < 0.001) and smokers (14.0% vs. 10.7%, *p* = 0.036). Hospitalised individuals had a higher prevalence of myocardial infarction (23.9% vs. 11.6%, *p* < 0.001) and other cardiac diseases (18.8% vs. 10.8%, *p* < 0.001) (Table S1). Additionally, peripheral vascular disease (22.0% vs. 13.0%, *p* < 0.001), cancer (25.1% vs. 18.8%, *p* = 0.002), and asthma/COPD (25.5% vs. 19.3%, *p* = 0.002) and joint diseases, were more common among hospitalised participants. They also rated their health lower than not-hospitalised participants. Hospitalised participants had lower education levels (22.6% vs. 17.6%, *p* = 0.002) and lower income. Although household size and living arrangements were similar, hospitalised participants reported more loneliness on the loneliness scale of de Jong-Gierveld (4.1 ± 3.7 vs. 3.7 ± 3.5, *p* = 0.005) and less access to informal care (20.1% vs.13.3%, *p* < 0.001).

**Table 2:** Baseline characteristics: hospitalised versus not-hospitalised participants

		Total *n* = 1803	Not-hospitalised *N* = 967	Hospitalised *N* = 836	*p*-value
Age, years		75.8 ± 6.5	75.6 ± 6.4	76.1 ± 6.6	0.100
Gender	Male	707 (39.2%)	325 (33.6%)	382 (45.7%)	< 0.001
	Female	1096 (60.8%)	642 (66.4%)	454 (54.3%)	
BMI (kg/m^2^), continuous		26.8 (24.1–29.5)	26.6 (24.0–29.4)	26.9 (24.2–29.7)	0.376
Marital status	Married/living together	526 (30.1%)	280 (30.0%)	246 (30.3%)	0.886
	Unmarried/divorced/widowed	1220 (69.9%)	654 (70.0%)	566 (69.7%)	
Frailty, according to Fried	Pre-frail	1456 (87.7%)	834 (86.2%)	622 (25.6%)	< 0.001
	Frail	347 (12.3%)	133 (13.8%)	214 (74.4%)	
Smoking	Yes	218 (12.1%)	102 (10.7%)	116 (14.0%)	0.036
	No	1558 (86.4%)	847 (89.3%)	711 (86.0%)	
Alcohol	Yes	1234 (71.5%)	671 (72.2%)	563 (70.6%)	0.442
	No	493 (28.5%)	258 (27.8%)	235 (29.4%)	
Number of chronic diseases	0 or 1	288 (16.0%)	175 (18.1%)	113 (16.0%)	0.008
	2 or more	1515 (84.0%)	792 (81.9%)	723 (86.5%)	
Level of education	High	232 (13.5%)	146 (15.6%)	86 (10.9%)	0.002
	Medium	1148 (66.7%)	624 (66.8%)	524 (66.5%)	
	Low	342 (19.9%)	164 (17.6%)	178 (22.6%)	
Income	> €31.000	200 (11.1%)	130 (13.5%)	70 (8.4%)	< 0.001
	Max €31.000	310 (17.2%)	180 (18.6%)	130 (15.6%)	
	Max €24.000	458 (25.4%)	249 (25.8%)	209 (25.0%)	
	Max €19.400	646 (35.8%)	319 (33.0%)	327 (39.1%)	
	Max €15.200	188 (10.4%)	88 (9.1%)	100 (12.0%)	
Household size		2 (1–2)	2 (1–2)	2 (1–2)	0.717
Living situation	Not living alone	645 (36.8%)	348 (36.9%)	297 (36.7%)	0.905
	Living alone	1107 (63.2%)	594 (63.1%)	513 (63.3%)	
Self-perceived health	Very good or good	572 (32.6%)	380 (40.4%)	192 (23.6%)	< 0.001
	Fair	962 (54.8%)	495 (52.7%)	467 (57.3%)	
	Poor or very poor	221 (12.6%)	65 (6.9%)	156 (19.1%)	
Social network type	Private restricted	157 (9.5%)	75 (10.7%)	82 (10.7%)	0.190
	Family dependent	359 (21.8%)	178 (20.3%)	181 (23.6%)	
	Locally integrated	606 (36.8%)	335 (38.2%)	271 (35.3%)	
	Local self-contained	401 (24.4%)	219 (24.9%)	182 (23.7%)	
	Wider community focused	123 (7.5%)	71 (8.1%)	52 (6.8%)	
Availability of informal care, no		297 (16.5%)	129 (13.3%)	168 (20.1%)	< 0.001
Loneliness-scale, de Jong-Gierveld		3.9 ± 3.6	3.7 ± 3.5	4.1 ± 3.7	0.005

BMI: Body Mass Index. Data are reported as number of participants (%), mean ± standard deviation or median (Q1-Q3). Percentages were computed relative to the total number of participants in the presented column. Some baseline data was missing: BMI 5.2%, marital status 3.2%, household size 2.8%, education 4.5%, alcohol 4.2%, smoking 1.5%, SPH 2.7%, loneliness 4.6%

#### Comparison of hospitalised versus not-hospitalised participants within frail and pre-frail groups

Results of the baseline characteristics of the participants, divided into frail and pre-frail groups, are detailed in the supplementary appendix (Table S2). Among frail individuals, hospitalised participants had higher rates of male gender and lower BMI compared to not-hospitalised ones. Notably, 94.6% of the frail participants rated their SPH as fair, poor or very poor with lower scores and more comorbidities (non-significant) in the hospitalised group. Within pre-frail participants the higher prevalence of male gender and lower SPH persisted, accompanied by additional differences: more chronic diseases, lower educational level, lower income, and less availability of informal care in the hospitalised group.

### Odds ratio’s for hospitalisation at 24 months follow-up

#### Multivariable analysis: all pre-frail and frail participants

First, we conducted a univariable analysis of all participant characteristics, with the results provided in the supplementary appendix (Table S3). Seven factors were found to be associated with hospitalisation in pre-frail and frail participants. Male gender (OR 1.66, 95% CI 1.37–2.01, *p* < 0.001), being frail (OR 2.16, 95% CI 1.70–2.74, *p* < 0.001), 2 or more chronic diseases (OR 1.41, 95% CI 1.09–1.83, *p* = 0.008), lower income (max €24.000 OR 1.56, 95% CI 1.11–2.20, *p* = 0.011), fair (OR 1.87, 95% CI 1.51–2.31, *p* < 0.001) or poor to very poor (OR 4.75, 95% CI 3.39–6.66, *p* < 0.001) SPH, lack of informal care (OR 1.63, 95% CI 1.27–2.10, *p* < 0.001) and higher levels of experienced loneliness on the loneliness-scale (OR 1.04, 95% CI 1.01–1.07, *p* = 0.005) were significantly associated with hospitalisation. The multivariable analysis, with results presented in Table 3, confirmed being male (OR 1.65, *p* < 0.001), frail (OR 1.66, *p* = 0.002), SPH assessed as fair or poor-very poor (respectively OR 1.42, *p* = 0.008 and OR 3.12, *p* < 0.001), and lack of informal care (OR 1.69, *p* < 0.001) to be independently associated with hospitalisation.

**Table 3:** Stepwise backward logistic regression for hospitalisation, all participants

		Multivariable analysis (*n* = 1803)		
		OR	95% CI	p-value
Gender, male		1.65	1.31–2.08	< 0.001
Frailty, frail		1.66	1.20–2.28	0.002
Self-perceived health	Very good or good Fair Poor or very poor	Ref. 1.42 3.12	2.05–4.73 1.10–1.83	0.008 < 0.001
Availability of informal care, no		1.69	1.24–2.31	< 0.001

Results are not adjusted

#### Multivariable analysis: sub analysis of frail and pre-frail participants

Factors associated with hospitalisation varied between pre-frail and frail participants (Table 4). Due to the limited sample size of frail participants, it was not feasible to incorporate all predictors into the multivariable analysis. Therefore, the variables that exhibited significant associations within univariable analysis (table S4) (male gender (OR 2.29, 95%CI 1.42–3.68, *p* < 0.001), BMI (OR 0.94, 95% CI 0.89–0.99, *p* = 0.010, lower income (max €19.400 OR 2.88, 95% CI 1.09–7.60, *p* = 0.032), SPH (fair OR 6.67, 95% CI 1.87–23.77, *p* = 0.003; poor to very poor OR 16.43, 95% CI 4.43–60.87, *p* < 0.001), along with age, chronic diseases, marital status, social network, and availability of informal care (due to their observed associations in the pre-frail and frail total participants group), were included in the multivariable logistic regression. The multivariable analysis showed significant associations between hospitalisation and the male gender, lower income, lower SPH, social networks characterised by limited support and lack of informal care (Table 4). BMI was found to have a protective effect for hospitalisation in this subgroup.

In the univariable analysis, pre-frail participants showed comparable significant associations to frail participants in relation to male gender (OR 1.62, 95% CI 1.31–2.01,*p* < 0.001), income (max €24.000 OR 1.49, 95% CI 1.03–2.15, *p* = 0.034) and SPH (fair OR 1.67, 95% CI 1.33–2.09, *p* < 0.001; poor to very poor OR 3.34, 95% CI 2.15–5.19, *p* < 0.001) (Table S4). Additionally, a lower level of education (OR 1.70, 95% CI 1.16–2.49, *p* = 0.006) and lack of informal care (OR 1.59, 95% CI 1.19–2.13, *p* = 0.002) were significantly associated with hospitalisation. In the multivariable analysis (Table 4), male gender, lower SPH and lack of informal care remained independently associated with hospitalisation. Notably, BMI and smoking were also associated with hospitalisation in the multivariable model.

**Table 4:** Stepwise backward logistic regression for hospitalisation, subdivided into pre-frail and frail participants

		Frail participants (*n* = 347)			Pre-frail participants (*n* = 1456)		
		**Multivariable analysis**			**Multivariable analysis**		
		**OR**	**95% CI**	**p-value**	**OR**	**95% CI**	**p-value**
Gender, male		1.72	0.93–3.12	0.085	1.61	1.25–2.07	< 0.001
BMI (kg/m^2^), continuous		0.89	0.83–0.95	< 0.001	1.03	1.00–1.06	0.074
Smoking, yes					1.38	0.94–2.01	0.099
Income	> €31.000 Max €31.000 Max €24.000 Max €19.400 Max €15.200	Ref. 4.13 4.51 7.24 9.43	0.89–19.29 1.05–19.40 1.78–29.55 1.83–48.70	0.040 0.071 0.043 0.006 0.007			
Self-perceived health	Very good or good Fair Poor or very poor	Ref. 8.54 31.16	2.05–35.54 6.88–141.08	< 0.001 0.003 < 0.001	Ref. 1.38 2.23	1.06–1.80 1.36–3.64	0.017 0.001
Social network type (%)	Wider community focused Locally integrated Local self-contained Family dependent Private restricted	Ref. 7.30 6.16 6.73 10.74	2.01–26.58 1.70–22.36 1.83–24.76 2.42–47.60	0.025 0.003 0.006 0.004 0.002			
Availability of informal care, no		2.63	1.23–5.60	0.012	1.64	1.16–2.32	0.005

BMI: Body Mass Index. Results are not adjusted

## Discussion

This study aims to bridge the gap between medical and psychosocial factors in predicting hospitalisation by identifying individual factors within a pre-frail and frail population. Hospitalised participants were more often male, frail and smokers, experienced a higher burden of chronic diseases and had a lower educational attainment, lower income and lower SPH in comparison to not-hospitalised participants. Multivariable logistic analysis showed significant associations indicating higher odds for hospitalisation and the male gender, frailty (vs. pre-frailty), lower SPH and lack of informal care. These associations persisted in the subgroup analysis of pre-frail and frail participants. Additionally, among frail participants social network types characterised by limited support and lower income showed higher odds of hospitalisation. Conversely, BMI was found to have a protective effect in this sub-group.

Extensive research has been conducted on risk factors associated with hospitalisation in older adults. Medical and health-related factors as higher age, presence of chronic conditions, such as chronic heart failure or chronic obstructive pulmonary disease, and concurrent polypharmacy are significant factors influencing hospital rates [19–21]. Furthermore, frailty has been increasingly recognised as an important predictor of hospitalisation [13]. Frailty is associated with higher age, female gender, higher rates of chronic diseases, lower SPH, lower education and lower income [7]. However, the precise mechanisms that underlie the associations between frailty and the subsequent increased risk of hospitalisation remains unknown. Comprehending these associations and risk factors is imperative for effective health care planning and mitigating adverse outcomes, particularly hospitalisation, among a globally ageing population.

The objective of our study was to identify additional individual factors within a pre-frail and frail population. In contrast to the commonly observed association of frailty with the female gender and older age, this study showed a significant association with male gender and no association with age, suggesting that individual predictive factors may differ within a pre-frail and frail population [7].

The results also underline the important association between SPH and hospitalisation. Lower SPH, often concurrent with a higher number of comorbidities, is associated with presence of frailty and identified as a predictor of mortality and other negative health outcomes, such as hospitalisation and disability. It has been described as a useful concept in prevention [15, 22–24]. This study also showed that hospitalised participants experienced a higher burden of chronic diseases and lower scores of SPH. Additionally, it demonstrated that within a pre-frail and frail population, where approximately 70% of the participants rated their health as fair to very poor, lower SPH remained a discriminative factor for hospitalisation, highlighting the importance of subjective health assessment. Furthermore, social characteristics such as higher levels of loneliness and lack of informal care exhibited a strong association for hospitalisation in pre-frail and frail participants. This aligns with the understanding that frailty is a multidimensional concept encompassing psychological and social dimensions, whose impact on adverse health outcomes remains under recognised [25]. Research shows social network is an important factor in persons with frailty. A meta-analysis of the effect of social relationships on mortality found that people with adequate relationships, assessed through various measures including marital status and social network types, were 50% more likely to survive [26]. Examining the longitudinal relationship between physical and social domains of frailty has shown that loneliness and social isolation may contribute to the onset of physical frailty [27]. This emphasizes the persistent associations between hospitalisation and loneliness as well as lack of available informal care found in this study. Additionally, implementing social support networks in a pre-frail and frail population of community dwelling older persons demonstrated that social support can lead to improved frailty symptoms [28].

The protective effect of high BMI on hospitalisation found in frail older participants, could be in line with the so-called ‘obesity-paradox’ [29]. This term refers to the counter-intuitive observation that older adults who are overweight or obese may have better outcomes for certain diseases compared to those with normal or lower body weight. As body composition naturally changes with advancing age, often leading to weight loss, it has been proposed that higher body fat levels in older adults may actually confer health benefits.

Overall, while literature shows factors as age and chronic diseases contribute both to frailty and hospitalisation, the results of this study suggest socio-demographic factors appear to have a more pronounced impact in a population of pre-frail and frail community-dwelling older people. Being male, frail (vs. pre-frail), experiencing lower SPH and lack of informal care surface as important determinants for hospitalisation. Furthermore, frailty is a dynamic state among older persons and transitions to states of greater frailty are common following hospitalisation [30]. As a result of various factors during hospitalisation such as bed restriction, delirium or falls, older pre-frail individuals often do not return to baseline functional status and experience increased risk of institutionalisation, hospital readmissions and deaths [31, 32]. Given these implications, particular attention should be directed towards pre-frail individuals, as interventions targeting these subgroups hold significant potential for beneficial outcomes.

### Strengths and limitations

The strength of the current study include the prospective collection of data from a substantial sample of community-dwelling older individuals classified as pre-frail and frail, coupled with a follow-up period spanning 24 months. However, the study has several limitations. The absence of baseline data, particularly for characteristics such as BMI and social network type, constrains the analysis, although the percentage of missing data is low. Moreover, a significant portion of hospitalisation outcome data was missing. Participants with missing data were often frail, older, and living alone, and tended to have worse baseline scores, which calls for cautious interpretation of the findings. Selective mortality or admission to a nursing home may have also influenced the results, as the most severely frail participants were more likely to drop out for these reasons. Nevertheless, the potential for recall bias is considered minimal, given that individuals are unlikely to forget a significant event like hospitalisation within the past six months. Lastly, it is important to note that the statistical techniques used in this study do not allow for causal inferences.

## Conclusion

This study provides insight in the individual factors related to hospitalisation among a pre-frail and frail older population and highlight the fundamental role of psychosocial factors on adverse outcomes. Identifying psychosocial risk-factors can improve risk stratification for older participants and suggest targeted interventions. For example, enhancing social support networks and increasing the availability of informal care in this population might lead to reduced hospitalisation rates. Further research should focus on evaluating individual risk-factors for hospitalisation and further explore the psychosocial determinants associated with adverse outcomes in an older population. Particularly, attention should be directed towards pre-frail individuals, as preventing progression to frailty holds significant potential for beneficial outcomes.

In conclusion, these findings highlight the importance of understanding both medical and psychosocial determinants of frailty to inform effective health care planning and subsequently intervention strategies.

## Electronic supplementary material

Below is the link to the electronic supplementary material.

Supplementary Material 1

## Data Availability

The datasets generated for and analysed during the current study are not publicly available. Nonetheless, data are available from the corresponding author upon reasonable request.

## Abbreviations

95% CI: 95% confidence interval
BMI: Body mass index
IQR: Interquartile range
OR: Odds ratio
SD: Standard deviation
SPH: Self-perceived health
