# Supplementary material for: Risk factors for hospitalisation in community-dwelling pre-frail and frail older people: results of a longitudinal study
Source: BMC Geriatr. 2024 Oct 19;24:850. doi: 10.1186/s12877-024-05458-4 (PMC11490166; doi:10.1186/s12877-024-05458-4)
Supplement: Supplementary file 1 — Supplementary Material 1 [file 12877_2024_5458_MOESM1_ESM.docx]

**Appendix**

**Table S1. Chronic diseases**

*Data are reported as number of participants (%).*

|  | Total  n = 1803 | Frail  N = 347 | Pre—frail  N = 1456 | p-value | Not-hospitalised  N = 967 | Hospitalised  N = 836 | p-value |
| --- | --- | --- | --- | --- | --- | --- | --- |
| Diabetes | 385 (22.6%) | 96 (28.9%) | 289 (21.0%) | 0.002 | 193 (21.1%) | 192 (24.3%) | 0.117 |
| Stroke/cerebral haemorrhage/cerebral infarction | 256 (15.1%) | 68 (21.1%) | 188 (13.7%) | < 0.001 | 125 (13.7%) | 131 (16.8%) | 0.077 |
| Myocardial infarction | 292 (17.3%) | 91 (27.8%) | 2012 (14.8%) | < 0.001 | 106 (11.6%) | 186 (23.9%) | < 0.001 |
| Other cardiac diseases | 247 (13.7%) | 65 (19.8%) | 182 (13.3%) | 0.002 | 99 (10.8%) | 148 (18.8%) | < 0.001 |
| Hypertension | 785 (46.7%) | 165 (50.2%) | 620 (45.9%) | 0.165 | 429 (47.4%) | 356 (46.0%) | 0.579 |
| Peripheral vascular disease | 284 (17.1%) | 84 (25.8%) | 200 (15.0%) | < 0.001 | 117 (13.0%) | 167 (22.0%) | < 0.001 |
| Cancer | 373 (21.7%) | 78 (23.7%) | 295 (21.2%) | 0.319 | 174 (18.8%) | 199 (25.1%) | 0.002 |
| Asthma or chronic obstructive pulmonary disease | 370 (22.1%) | 92 (28.2%) | 278 (20.6%) | 0.003 | 175 (19.3%) | 195 (25.5%) | 0.002 |
| Hip or knee arthrosis | 1037 (60.7%) | 240 (71.2%) | 797 (58.1%) | < 0.001 | 535 (58.0%) | 502 (63.9%) | 0.012 |
| Chronic joint inflammation | 465 (27.9%) | 131 (40.6%) | 334 (24.9%) | < 0.001 | 232 (25.6%) | 233 (30.7%) | 0.022 |
| Back problems (including hernia) | 539 (29.9%) | 144 (43.8%) | 395 (29.0%) | < 0.001 | 265 (29.0%) | 274 (35.3%) | 0.006 |

**Table S2. Baseline characteristics: hospitalised versus not-hospitalised participants within frail and pre-frail groups**

*Values on some baseline characteristics were missing, percentages are reported. Data are reported as number of participants (%), mean ± standard deviation or median (Q1-Q3). Percentages in the ‘total’ column are of total number of participants. Percentages within the hospitalised and not-hospitalised groups are calculated based on the total number of participants in the specific column. Some baseline data was missing: BMI 5.2%, marital status 3.2%, household size 2.8%, education 4.5%, alcohol 4.2%, smoking 1.5%, SPH 2.7%, loneliness 4.6%.*

|  |  | FRAIL PARTICIPANTS | | | | PRE-FRAIL PARTICIPANTS | | | |
| --- | --- | --- | --- | --- | --- | --- | --- | --- | --- |
|  |  | **Total**  **N = 347** | **Not-hospitalised (n = 133)** | **Hospitalised**  **(n = 214)** | **p-value** | **Total**  **N = 1456** | **Not-hospitalised (n = 834)** | **Hospitalised**  **(n = 622)** | **p-value** |
| Age, years | | 77.6 ± 6.7 | 78.5 ± 6.4 | 77.1 ± 7.0 | 0.067 | 75.4 ± 6.4 | 75.2 ± 6.3 | 75.8 ± 6.5 | 0.064 |
| Gender | Male  Female | 125 (36.0%)  222 (64.0%) | 33 (24.8%)  100 (75.2%) | 92 (43.0%)  122 (57.0%) | < 0.001 | 582 (40.0%)  874 (60.0%) | 292 (35.0%)  542 (65.0%) | 290 (46.6%)  332 (53.4%) | < 0.001 |
| BMI (kg/m^2^), continuous | | 27.1 (24.2–30.0) | 28.1 (24.5–31.1) | 26.7 (24.1–29.1) | 0.019 | 26.7 (24.0–29.4) | 26.4 (23.9–29.3) | 26.9 (24.2–29.8) | 0.059 |
| Marital status | Married/living together  Unmarried/divorced/ widowed | 112 (33.4%)  223 (66.6%) | 44 (34.6%)  83 (65.4%) | 68 (32.7%)  140 (67.3%) | 0.713 | 414 (29.3%)  997 (70.7%) | 236 (29.2%)  571 (70.8%) | 178 (29.5%)  426 (70.5%) | 0.926 |
| Smoking | Yes  No | 56 (16.3%) 287 (83.7%) | 19 (14.5%)  112 (85.5%) | 37 (17.5%)  175 (82.5%) | 0.473 | 162 (11.3%)  1271 (88.7%) | 83 (10.1%)  735 (89.9%) | 79 (12.8%)  536 (87.2%) | 0.110 |
| Alcohol | Yes  No | 194 (59.9%)  120 (40.1%) | 72 (59.0%)  50 (41.0%) | 122 (60.4%)  80 (39.6%) | 0.806 | 1040 (74.1%)  363 (25.9%) | 599 (74.2%)  208 (25.8%) | 441 (74.0%)  155 (26.0%) | 0.922 |
| Number of chronic diseases | 0 or 1  2 or more | 25 (7.2%)  322 (92.8%) | 13 (9.8%)  120 (90.2%) | 12 (5.6%)  202 (94.5%) | 0.144 | 263 (18.1%)  1193 (81.9%) | 162 (19.4%)  672 (80.6%) | 101 (16.2%)  521 (83.8%) | 0.118 |
| Level of education | Low  Medium  High | 108 (32.9%)  202 (61.6%)  18 (5.5%) | 42 (33.3%)  77 (61.1%)  7 (5.6%) | 66 (32.7%)  125 (61.9%)  11 (5.4%) | 0.990 | 234 (16.8%)  946 (67.9%)  214 (15.4%) | 122 (15.1%)  547 (67.7%)  139 (17.2%) | 112 (19.1%)  399 (68.1%)  75 (12.8%) | 0.023 |
| Income | > €31.000  Max €31.000  Max €24.000  Max €19.400  Max €15.200 | 19 (5.5%)  48 (13.8%)  78 (22.5%)  161 (46.4%)  41 (11.8%) | 11 (8.3%)  22 (16.5%)  35 (26.3%)  52 (39.1%)  13 (9.8%) | 8 (3.7%)  26 (12.1%)  43 (20.1%)  109 (50.9%)  28 (13.1%) | 0.064 | 181 (12.4%)  262 (18.0%)  380 (26.1%)  485 (33.3%)  147 (10.1%) | 119 (14.3%)  158 (19.0%)  214 (25.7%)  267 (32.1%)  75 (9.0%) | 62 (10.0%)  104 (16.7%)  166 (26.7%)  218 (35.0%)  72 (11.6%) | 0.045 |
| Household size | | 2.0 (1.0–2.0) | 2.0 (1.0–2.0) | 2.0 (1.0–2.0) | 0.943 | 2.0 (1.0–2.0) | 2.0 (1.0–2.0) | 2.0 (1.0–2.0) | 0.459 |
| Living situation | Living alone  Not living alone | 136 (40.8%)  197 (59.2%) | 50 (39.7%)  76 (60.3%) | 121 (58.5%)  86 (41.5%) | 0.737 | 509 (35.9%)  910 (64.1%) | 298 (36.5%)  518 (63.5%) | 211 (35.0%)  392 (65.0%) | 0.553 |
| Self-perceived health | Very good or good  Fair  Poor or very poor | 18 (5.4%)  196 (58.7%)  120 (35.9%) | 15 (11.8%)  84 (66.1%)  28 (22.0%) | 3 (1.4%)  112 (54.1%)  92 (44.4%) | < 0.001 | 554 (39.0%)  766 (53.9%)  101 (7.1%) | 365 (44.9%)  411 (50.6%)  37 (4.6%) | 189 (31.1%)  355 (58.4%)  64 (10.5%) | < 0.001 |
| Social network type | Private restricted  Family dependent  Locally integrated  Local self-contained  Wider community focused | 48 (15.2%)  77 (24.4%)  100 (31.6%)  73 (23.1%)  18 (5.7%) | 17 (14.0%)  28 (23.1%)  38 (31.4%)  26 (21.5%)  12 (9.9%) | 31 (15.9%)  49 (25.1%)  62 (31.8%)  47 (24.1%)  6 (3.1%) | 0.156 | 109 (8.2%)  282 (21.2%)  506 (38.0%)  328 (24.7%)  105 (7.9%) | 58 (7.7%)  150 (19.8%)  297 (39.2%)  193 (25.5%)  59 (7.8%) | 51 (8.9%)  132 (23.0%)  209 (36.5%)  135 (23.6%)  46 (8.0%) | 0.497 |
| Availability of informal care, no | | 80 (23.1%) | 26 (19.5%) | 54 (25.2%) | 0.222 | 217 (14.9%) | 103 (12.4%) | 114 (18.3%) | 0.002 |
| Loneliness-scale, de Jong-Gierveld | | 5.0 (2.0–8.0) | 5.0 (2.0–8.0) | 5.0 (2.0–9.0) | 0.368 | 3.61 ± 3.51 | 3.47 ± 3.43 | 3.79 ± 3.60 | 0.100 |

**Table S3. Univariable analysis for hospitalisation, all participants.**

*BMI: Body Mass Index. Results are not adjusted.*

|  |  | PRE-FRAIL AND FRAIL PARTICIPANTS | | |
| --- | --- | --- | --- | --- |
|  |  | **Univariable analysis** | | |
|  |  | **OR** | **95% CI** | **p-value** |
| Age, years | | 1.01 | 1.00–1.03 | 0.100 |
| Gender, male | | 1.66 | 1.37–2.01 | < 0.001 |
| BMI (kg/m^2^), continuous | | 1.01 | 0.99–1.03 | 0.432 |
| Marital status | Married/living together  Unmarried/divorced/Widowed | Ref.  0.99 | 0.80–1.20 | 0.886 |
| Frailty | Pre-frail  Frail | Ref.  2.16 | 1.70–2.74 | < 0.001 |
| Smoking, yes | | 1.36 | 1.02–1.80 | 0.036 |
| Alcohol, yes | | 0.92 | 0.75–1.14 | 0.442 |
| Number of chronic diseases | 0 or 1  2 or more | Ref.  1.41 | 1.09–1.83 | 0.008 |
| Level of education | High  Medium  Low | Ref.  1.43  1.84 | 1.07–1.91  1.31–2.59 | 0.017  < 0.001 |
| Income | > €31.000  Max €31.000  Max €24.000  Max €19.400  Max €15.200 | Ref.  1.34  1.56  1.90  2.11 | 0.93–1.94  1.11–2.20  1.37–2.65  1.40–3.17 | 0.118  0.011  < 0.001  < 0.001 |
| Household size | | 1.00 | 0.90–1.11 | 0.972 |
| Living situation, alone | | 0.99 | 0.81–1.20 | 0.905 |
| Self-perceived health | Very good or good  Fair  Poor or very poor | Ref.  1.87  4.75 | 1.51–2.31  3.39–6.66 | < 0.001  < 0.001 |
| Social network type (%) | Private restricted  Family dependent  Locally integrated  Local self-contained  Wider community focused | Ref.  0.93  0.74  0.76  0.67 | 0.64–1.35  0.52–1.05  0.53–1.10  0.42–1.08 | 0.705  0.093  0.146  0.099 |
| Availability of informal care, no | | 1.63 | 1.27–2.10 | < 0.001 |
| Loneliness-scale, de Jong-Gierveld | | 1.04 | 1.01–1.07 | 0.005 |

**Table S4. Univariable analysis for hospitalisation, subdivided into pre-frail and frail participants.**

*BMI: Body Mass Index. Results are not adjusted.*

|  |  | FRAIL PARTICIPANTS | | | PRE-FRAIL PARTICIPANTS | | |
| --- | --- | --- | --- | --- | --- | --- | --- |
|  |  | **Univariable analysis** | | | **Univariable analysis** | | |
|  |  | **OR** | **95% CI** | **p-value** | **OR** | **95% CI** | **p-value** |
| Age, years | | 0.97 | 0.94–1.00 | 0.068 | 1.02 | 1.00–1.03 | 0.064 |
| Gender, male | | 2.29 | 1.42–3.68 | < 0.001 | 1.62 | 1.31–2.01 | < 0.001 |
| BMI (kg/m^2^), continuous | | 0.94 | 0.89–0.99 | 0.010 | 1.03 | 1.00–1.05 | 0.059 |
| Marital status | Married/living together  Unmarried/divorced/Widowed | Ref.  1.09 | 0.69–1.74 | 0.713 | Ref.  0.99 | 0.79–1.25 | 0.926 |
| Smoking, yes | | 1.25 | 0.68–2.28 | 0.473 | 1.31 | 0.94–1.81 | 0.111 |
| Alcohol, yes | | 1.06 | 0.67–1.67 | 0.806 | 0.99 | 0.78–1.26 | 0.922 |
| Number of chronic diseases | 0 or 1  2 or more | Ref.  1.82 | 0.81–4.13 | 0.149 | Ref.  1.24 | 0.95–1.64 | 0.118 |
| Level of education | High  Medium  Low | Ref.  1.03  1.00 | 0.38 0 2.78  0.36–2.78 | 0.949  1.000 | Ref.  1.35  1.70 | 0.99–1.84  1.16–2.49 | 0.056  0.006 |
| Income | > €31.000  Max €31.000  Max €24.000  Max €19.400  Max €15.200 | Ref.  1.63  1.69  2.88  2.96 | 0.56–4.75  0.61–4.66  1.09–7.60  0.96–9.11 | 0.375  0.311  0.032  0.058 | Ref.  1.26  1.49  1.57  1.84 | 0.85–1.87  1.03–2.15  1.10–2.24  1.18–2.88 | 0.245  0.034  0.013  0.007 |
| Household size | | 1.03 | 0.78–1.35 | 0.846 | 1.01 | 0.90–1.13 | 0.909 |
| Living situation, alone | | 1.08 | 0.69–1.70 | 0.737 | 0.94 | 0.75–1.17 | 0.553 |
| Self-perceived health | Very good or good  Fair  Poor or very poor | Ref.  6.67  16.43 | 1.87–23.77  4.43–60.87 | 0.003  < 0.001 | Ref.  1.67  3.34 | 1.33–2.09  2.15–5.19 | < 0.001  < 0.001 |
| Social network type (%) | Private restricted  Family dependent  Locally integrated  Local self-contained  Wider community focused | Ref.  0.96  0.90  0.99  0.27 | 0.45–2.04  0.44–1.83  0.46–2.12  0.09–0.86 | 0.915  0.761  0.982  0.027 | Ref.  1.00  0.80  0.80  0.89 | 0.64–1.56  0.53–1.21  0.51–1.23  0.52–1.52 | 0.997  0.294  0.303  0.662 |
| Availability of informal care, no | | 1.39 | 0.82–2.36 | 0.223 | 1.59 | 1.19–2.13 | 0.002 |
| Loneliness-scale, de Jong-Gierveld | | 1.03 | 0.97–1.09 | 0.373 | 1.03 | 1.00–1.06 | 0.100 |
